# Current status of ablative therapies for renal tumors

**DOI:** 10.4103/0970-1591.57928

**Published:** 2009

**Authors:** Adam C. Mues, Jaime Landman

**Affiliations:** Columbia University, Department of Urology, 161 Fort Washington Avenue, Herbert Irving Pavilion, 11^th^ Floor, New York, NY 10032, USA

**Keywords:** Small renal mass, renal cryoablation, radiofrequency ablation

## Abstract

The increase in detection of small (≤ 4 cm) renal cortical neoplasms has made nephron-sparing surgery the new standard of care for T1a renal lesions. Advances in minimally invasive surgery have improved the surgical approach to these lesions to include laparoscopic partial nephrectomy and renal ablative therapies. In this review, we discuss the indications, outcomes, and potential complications of the commonly used ablative modalities in urologic practice. We will expand on renal cryoablation and review the mechanism of action, surgical approaches, and evidence based medicine using this modality.

## INTRODUCTION

The incidence of renal cell carcinoma (RCC) is rising in the United States.[1] There has been an increase in detection of incidental small renal mass (≤ 4cm) cases due to widespread use of abdominal cross sectional imaging and thus an increase in the amount of renal surgery performed.[2–4] Although radical nephrectomy has been the gold standard for RCC treatment, nephron-sparing approaches such as open and laparoscopic partial nephrectomy have become the new standard for the treatment of clinical T1a renal lesions.[5] Advances in probe ablative technology have introduced new additional minimally invasive alternatives for the treatment of small renal masses (SRM) in the form of renal cyroablation and radiofrequency ablation (RFA). Laparoscopic partial nephrectomy (LPN) has demonstrated excellent outcomes in terms of oncologic control. However, some major disadvantages of the procedure are - technical challenges of parenchymal hemostasis, pelvicaliceal reconstruction and parenchymal renorrhaphy. These lead to longer warm ischemia times.[6,7] In addition, complication rates with nephron-sparing surgery have been reported to be higher compared with ablation procedures.[8] Ablation therapy was originally recommended in elderly patients, high-risk surgical candidates, and those who rejected surgical intervention. The advantages of renal ablation include a reduction in blood loss, shorter hospitalization, decreased postoperative pain, and reduction in complications when compared to laparoscopic partial nephrectomy. Desai reported that laparoscopic partial nephrectomy, in comparison with laparoscopic cryoablation, resulted in twice the blood loss (211 vs. 101mL) and an increase in rate of overall complications (32 vs. 6.7%).[8] Hence ablation has expanded as a viable option for treatment of small renal masses in all patients. Practice patterns in university based tertiary hospitals indicate that 93% of reporting academic urology centers offer ablation with a greater trend toward cryoablation over RFA.[9] Popularity for this modality in urology hinges on short-term patient benefits as well as certain advantages to the surgeon including accurate targeting and ablation of tumors, continuous visual and radiographic monitoring, and faster operative times compared to partial nephrectomy. Cryoablation can be performed successfully using open surgical, laparoscopic, and percutaneous approaches. However, to date there are only intermediate-term outcomes using cryoablation for renal tumors. Tumor destruction at the time of ablation is uncertain and oncologic efficacy and assessment of treatment is mainly evaluated with radiologic imaging parameters. In addition, long-term cancer control is unknown.

## PHYSIOLOGICAL EFFECTS ON TISSUE AND TUMOR

Cryoablation and its medical application began with the combined efforts of Dr. Irving Cooper, a neurosurgeon, and Arnold Lee, an engineer. The first cryosurgical system consisted of surface treatment with liquid nitrogen delivery through an insulated trocar and a metal tip which caused destruction of targeted tissue with limited damage to adjacent structures.[10,11] The first urologic application occurred with cryotherapy in the prostate for the treatment of benign prostatic hyperplasia and prostate cancer.[11] Subsequently, Uchida *et al*. performed the first cryoablation in the kidney in two human subjects.[12] Cryosurgery causes cell death by a number of different mechanism including intracellular ice formation and delayed microcirculatory failure. Rapid freezing results in an increased extracellular osmotic concentration causing fluid to be shifted from the intracellular space leading to a relative dehydration. Damage to the cell occurs due to changes in intracellular pH and protein denaturation as well as mechanical disruption of the cell membranes.[13] The thaw phase results in endothelial damage and cell death.

Delayed damage to the cell occurs over hours to days due to microvascular injury leading to widespread thrombosis, coagulative necrosis, and apoptosis. Modern cryoablation systems are composed of pressurized argon gas that is delivered to the cryoprobes. This system causes freezing through the Joule-Thomson principle, which occurs when rapid cooling results from a phase change of a highly compressed liquid expanding through a restricted orifice (cryoprobe) to a gaseous state. The tumoricidal temperature required for cell destruction has been shown to be −40C.[14] However, temperatures within a cryolesion are not uniform, but are variable with respect to the cryoprobe tip. Core temperatures at the probe tip fall between −140C and −190C with temperatures at the visible edge of the ice ball being 0C. In addition, a double freeze-thaw cycle significantly increased cell death compared to a single cycle (62 vs. 22% at 10 C and 89 vs. 63% at 15 C respectively). For each of the temperatures tested, extended freeze hold times and passive thawing rates have been shown to result in more extensive cell damage.[15] Two cycles have also been shown to produce a larger area of necrosis in an animal model compared to a single cycle.[16] However, the freeze time is not considered clinically significant.[17] Campbell and colleagues demonstrated that temperatures less than −20C can be achieved at a distance of 3.1 mm from the visible edge of the ice ball.[18] Therefore, most surgeons attempt to extend the cyroablation 1 cm beyond the lesion to ensure necrosis.

## SURGICAL APPROACHES WITH CRYOABLATION

### Open surgery

Rukstalis and co-workers first reported clinical experience with open renal cryoablation. They treated 29 patients with a median follow-up of 16 months. A subcostal incision was made followed by a transperitoneal approach to the kidney. A variable number of cryoprobes were placed directly into the mass and positioned using ultrasound so that the zone of ablation extended to a minimum of a 0.5 cm border. A double freeze-thaw cycle was used in all patients. One case was converted to radical nephrectomy involving a 2 cm solid mass found to invade the peri-renal fat on exploration. Patients were followed post-operatively with MRI scans. Five major adverse events occurred including one patient with biopsy proven RCC discovered at three month follow-up imaging, enhancement on MRI. The patient subsequently underwent a second open cryoablation. In addition, one patient suffered post-operative congestive heart failure and three patients required dialysis for chronic renal failure.[19]

### Laparoscopic cryoablation

Laparoscopic cryoablation (LCA) performed through a transperitoneal approach is generally used for anterior, antero-medial, and hilar renal masses. A retroperitoneal approach is used for posterior and lateral tumors. Laparoscopic mobilization of the kidney with complete visualization of the renal mass and hilum is valuable to the surgeon to visually monitor the procedure and assess early bleeding that may require additional intervention. Cryoprobes are placed under direct vision to precisely assess the location and depth of the probe and monitor the ice ball formation and ablation process.

### Patient positioning

The patient is initially supine so as to gain intravenous access as well as for the placement of a Foley catheter and an orogastric tube. Next, the patient is placed at a 45° angle in a modified lateral decubitus position, and the bed is flexed for adequate access to the kidney. An axillary role is placed and the patient's arm is secured. Care is taken to properly secure the patient with pillows, foam rolls and towels to ensure that all pressure points and joints are padded properly. The patient is taped into position with wide surgical tape and towels for stabilization and to facilitate table movement as required during the procedure. Core biopsies are taken as a routine part of surgery.

### Surgical approach

Laparoscopic access to the kidney is obtained by the formation of a pneumoperitoneum with the veress needle or Hasson technique and trocars are placed under direct vision. Transperitoneal access begins with an initial visual inspection of the abdominal cavity. The colon is mobilized and therefore, kidney is exposed by incising the white line of Toldt from the upper pole to the iliac vessels inferiorly. Next, the colon is reflected medially and the superior attachments are released to further expose the kidney (the splenorenal ligament is incised to mobilize the left kidney and the right triangular and anterior coronary ligaments are incised during a right sided procedure). Next, the psoas muscle, gonadal vessels, and ureter are located. The gonadal vein and ureter are elevated while the posterior plane between the kidney and psoas muscle is developed. Dissection is continued cephalad to the renal hilum. It is important to completely clear Gerota's fascia from the renal vessels and obtain adequate access to the hilum prior to proceeding with cryoablation in the event of excessive bleeding. Lesions may also be approached using a retroperitoneal approach. This is the preferred method for tumors that are posterior or lateral and not amenable to transperitoneal access. Next, the renal mass is targeted using pre-operative imaging and intra-operative ultrasound. The peri-renal fat is selectively removed from the capsule of the kidney to expose the surface of the lesion in its entirety and multiple core biopsies of the mass are taken using an 18-gauge percutaneous biopsy device.

Ultrasound is used to assess the depth of the lesion prior to probe placement. The cryoprobes (1.47mm) are placed percutaneously under direct vision into the abdominal cavity and guided into the renal mass using ultrasound. Cryoablation probes are deployed through the skin in such a manner that they enter the surface of the tumor at a 90 degree angle. Tumor size and shape decide the number of probes and the depth is assessed using ultrasound. Understanding the capabilities of the cryoablation probe used is also critical in deciding the number and configuration of probes for complete ablation. The first freeze cycle is performed followed by a thaw phase, which can be active, or passive and then a final freeze cycle. The ice ball is easily characterized on ultrasound as a hyperechoic rim with posterior acoustic shadowing. The rim increases in size during the freezing process and recedes during thawing. Intra-operative ultrasound ensures that the ice ball forms completely over the mass and extends 1cm beyond the gross margin of the tumor.[13] Removal of the probes should not be undertaken until adequate thawing occurs after the second freeze phase. The probes should twist completely with no resistance before removal. The lesion is monitored for hemorrhage with minor bleeding controlled with topical haemostatic agents such as FloSeal (Baxter, Glendale, CA) and gentle pressure. Following a final visual inspection of the ablation site at a reduced pneumoperitoneum, trocars are removed.

### Results

Measureable outcomes after cryoablation include reduction in size of tumor ablation site with no evidence of enhancement on post-operative imaging. Gill *et al*. evaluated 56 patients with a minimum of three-year follow-up after LCA. Biopsy of the ablation site was performed six months after the procedure. Recurrence of persistence of tumor occurred in two patients. The cancer specific survival at three years was 98%. Seventeen patients demonstrated complete disappearance of tumor.[20]

Weld and colleagues reported their three-year follow-up data on 31 patients after LCA. Transperitoneal and retroperitoneal approaches were used depending on location of the tumor and surgeon preference. Biopsy demonstrated that 61% of tumors were malignant and 39% benign or with indeterminate histopathology. One patient had radiographic enhancement suggestive of tumor during follow-up. However, the patient elected not to pursue biopsy due to several additional co morbidities. Cancer-specific survival was rated 100% and no patient developed metastatic disease.[21] Five year follow-up after LCA has been reported in 84 patients who underwent biopsy six months after the initial procedure. Four patient developed local recurrence alone, two patients had local recurrence and metastatic disease, and two had metastasis without local disease. Five-year overall and cancer-specific survival for patients with histologically proven RCC was 80 and 93% respectively.[22]

### Complications

Complications of LCA include hemorrhage, abscess formation, sepsis, ileus, renal insufficiency, paresthesia at the probe site, probe injury to adjacent organs and fistula formation. Significant bleeding is rare while fracture of the renal parenchyma is possible and has been reported to occur between 0-8.1%.[20,23,24]

Typically, bleeding from ablation site is managed with topical haemostatic agents; possibility of blood transfusion should, however, be discussed with the patient. Hruby and colleagues evaluated factors responsible for ice ball fracture in a porcine model; risk factors include premature removal of cryoprobes (before complete thawing), asynchronous ice ball formation (initiating a second ice ball after the primary ice ball has started to form), and specific ablation techniques used for upper pole lesions (guillotine technique). In this study, metrics tested included asynchronous ice ball formation using both small (1.47mm) and large (3.4mm) probes, synchronous formation using the 3.4mm probe, premature removal of the small (1.47mm) probe, and evaluation of the “guillotine technique” used to ablate upper pole renal tumors. The guillotine technique involves placement of three probes perpendicular to the kidney in a through and through manner with a fourth probe placed directly into the tumor. Asynchronous ice ball formation with the 1.47mm probe and premature removal of a probe did not result in hemorrhage. However, use of a 3.4mm probe to form the ice ball with a synchronous or asynchronous approach led to fracture and bleeding in 42 and 92% respectively.

Tumors ablated with the guillotine technique resulted in fracture in 54% of cases, which correlated with the group's clinical experience.[25] In a clinical evaluation, Lehman and colleagues compared results of LCA in patients with tumors less than 3 cm to those with tumors ≥ 3 cm. A significant difference was observed in the complication rate for patients with larger tumors (62%) compared with smaller tumors. The most frequent complication was hemorrhage requiring transfusion in 38%.[26]

### Percutaneous cryoablation

Percutaneous cryoablation (PCA) offers an additional minimally invasive treatment approach for ablation therapy. Posterior, lateral, and select anterior located tumors are indications for treatment with using a percutaneous approach.[26] Similar to the laparoscopic approach, PCA was first described in patients who were elderly, those considered to be poor surgical candidates based on co morbidities or previous abdominal surgery, and in patients with a solitary kidney. However, PCA is now an option for all patients with renal tumors ≤ 4cm in diameter. It is an important alternative for patients with renal insufficiency and those with hereditary RCC syndromes, susceptible to multiple and recurrent renal tumors.

### Advantages

- It can be performed on an outpatient basis
- Has been demonstrated to have significantly reduced post-operative pain
- Has a significant cost advantage compared with LCA[27]

### Disadvantages

- Lack of visual observation of the tumor during the ablation process
- Inability to assess immediate bleeding
- Mobilization of kidney is not possible and therefore probe placement is dependent on patient positioning to gain proper access to renal tumor

### Set-up and positioning

PCA can be performed in the operating room, but is more often done in a dedicated cystoscopy or radiology suite. The procedure is performed under conscious sedation or general anesthesia. Intravenous access is obtained prior to positioning and a Foley catheter is typically not required. The patient is placed in prone position with a targeting template on the ipsilateral flank and a CT scan is performed to correlate skin and renal anatomy. In certain cases the colon or small bowel is in close proximity to the renal tumor making percutaneous access dangerous. In this situation, repositioning the patient flank may alter the anatomy of the bowel making access possible.

An adequate distance between bowel structures, the ureter or other critical structures is confirmed by a repeat CT scan. Another technique used in repositioning failures is placement of a 5F catheter percutaneously along the colon in order to inject saline in an attempt to displace the bowel from the kidney. This procedure is often referred to as “floating” the bowel. Once safe access to the kidney has been assured, we deploy needle “access sheaths” (oscteocut needles) under CT guidance just outside the tumor and kidney. By deploying these access sheaths within Gerota's fascia and just outside the tumor, the access sheath now moves with the kidney and assures that subsequent biopsies and cryoablation needle deployment can be done without further CT scanning.

### Surgical technique

Once confirmation is obtained that the bowel and other abdominal organs have been safely shifted away from the kidney, and the access sheaths have been deployed, percutaneous biopsies are obtained. Cryoprobes are then deployed through the same access sheaths and a final CT scan is done to confirm that the needles are in perfect position and at the appropriate depth (just beyond deep margin of the tumor). A standard double freeze-thaw cycle is performed. The ice ball geometry and extension are monitored with ultrasound, CT, or MRI. In case the tumor cannot be approached safely, the procedure is aborted and plans are made for a laparoscopic cryoablation. Although ultrasound is an adequate imaging modality, it is not as precise as intra-operative sonography during LCA due to intervening structures (ribs and lungs).

In addition, the attenuation of the ice may limit clarity of the image and affect monitoring of the tumor during ablation. The CT is reasonably effective at discriminating frozen and unfrozen tissue and can accurately visualize the ice ball and monitor the extent of its formation as well as the tumor during the ablation process. After ablation is completed, we administer a half dose of intravenous contrast and perform a cortical phase CT scan to confirm adequacy of the ablation.

Ideally, the procedure is performed under sedation obviating the need for a general anesthetic. The patient avoids complications related to general anesthesia and surgical extirpation such as warm ischemia with vascular clamping. Additional benefits include a shorter hospitalization, reduction in pain medication requirements, and cost effectiveness.

### Results

Long-term follow-up for patients undergoing PCA is lacking at this time. However, small series demonstrate initial technical success as well as short-term patient benefits. Silverman and colleagues ablated twenty-six renal tumors with mean size of 2.6cm.

Patients received an MRI 24 hours following the procedure and at three-month intervals for the first year and six-month intervals thereafter. Mean follow-up was 14 months. Success was defined as no enhancement at the tumor site. Twenty-four of twenty-six tumors were successfully ablated with one procedure. One patient required a post procedure blood transfusion for a fall in hematocrit that subsequently normalized. A second patient's procedure was complicated by an abscess formation leading to a fistula between colon and the collecting system. Twenty-two of 26 patients were discharged on post-operative day (POD) 1.[28] In a large retrospective series of 115 tumors managed with PCA, patients were evaluated with post-procedure CT with and without IV contrast or MRI on POD 1. Mean tumor size was 3.3cm including 25% of tumors over 4cm. Technical success (no enhancement after procedure) occurred in 97% of cases and 87% of patients were discharged POD 1. Patients were imaged at three, six, and 12 months after PCA and then annually. There was no evidence of local progression (new enhancement or growth of the ablation site) in 80 tumors that were followed for a mean of 13.3 months.[29]

Patients with anterior tumors may present a challenge during PCA due to the proximity of adjacent organs. Most centers with extensive experience in PCA suggest that, technically and clinically, the procedure gives optimal results when performed by urologists and interventional radiologists. Urologists are essential for their expertise in renal anatomy, pathophysiology of renal tumors, and post-operative treatment and follow-up. In addition, the urologist has a surgical skill set necessary to solve procedure related problems and complications. Radiologists provide skill related to their experience with cross sectional imaging and accurate targeting of the renal lesions. Both specialists are valuable and necessary for the treatment of small renal masses using PCA.[27,29]

### Complications

Although complications with PCA are rare it is important to be familiar with potential problems with this approach. Major complications include inadequate oncologic treatment, injury to adjacent organs, collecting system injury contributing to fistula formation and active bleeding requiring transfusion. Minor complications include transient hematuria, pain, and hematoma formation. In a multi-institutional review, 20 complications were reported for PCA (16) and LCA (4). The most common issue reported was probe site pain or paresthesia. In addition, urinary tract infection, pneumonia, minor hemorrhage, and elevated creatinine occurred. One patient required reoperation for control of significant hemorrhage.[30]

## LAPAROSCOPIC VERSUS PERCUTANEOUS CRYOABLATION

Direct comparison of PCA and LCA based on technical aspects, patient outcomes, and complications has been reported. Finley and colleagues retrospectively reviewed the single center experience for cryoablation of 43 renal tumors (24 tumors treated with LCA and 19 treated with PCA). Percutaneous procedures were monitored with CT fluoroscopy and saline infusion was performed in 58% of cases.

PCA was superior to LCA with respect to operative time, hospital stay, narcotic requirement, and complication rate including transfusion (11.1% vs. 27.8). Hemorrhage was only associated with the use of multiple probes. Follow-up consisted of imaging with CT scan with IV contrast at six-month intervals. Short-term follow-up (median 11.4 for PCA and 13.4 for LCA) demonstrated a treatment failure rate of 5.3% for PCA and 4.7% for LCA. Cancer specific survival was 100% in both groups.[31] PCA has been associated with a shorter hospital stay, shorter anesthesia time and earlier return to nonstrenuous, strenuous activity. Malcom retrospectively reviewed 66 patients with a mean follow-up of 30 months after cryoablation. Fifty-two lesions were treated with LCA and 20 with PCA. Patients were followed with contrast enhanced CT scans at regular intervals with an increase in tumor size or persistent enhancement considered as failures. Re-treatment was offered in cases of failure with either repeat cryotherapy or extirpation. There were two (3.8%) primary treatment failures in the LCA group and five (25%) failures in the PCA group. Cancer-specific and cancer-free survival was 100% and 97% respectively. Complications included persistent ileus in two patients and the need for a blood transfusion in two patients (all occurred in LCA group).[32] Despite excellent oncologic control at 2.5 years, persistent follow-up is necessary and the potential for re-treatment should be communicated to all patients considering this form of treatment.

## SPECIAL CIRCUMSTANCES

### Multiple renal tumors

Lin reported a retrospective comparison of LCA and LPN in 27 patients with multiple ipsilateral renal tumors. Thirty-one tumors were treated with LCA and 28 with LPN. Patients in the LPN group had fewer tumors, larger tumors, and lower serum creatinine values pre-operatively compared to the LCA group. Renal functional outcomes measured were serum creatinine and estimated glomerular filtration rate (GFR). For patients undergoing LPN, the warm ischemia time was 36 minutes with two patients receiving ice-slush hypothermia and a cold ischemia time of 40 minutes.

Mean cryo-time was 13.5 minutes per lesion. Patients in the LPN group had greater blood loss and longer hospital stays compared to LCA. Complication rates, median operating times, and renal functional parameters were comparable in the two groups. After a follow-up of 38.5 and 24 months for the LPN and LCA groups respectively, the overall and cancer specific survival rates were both 100% for the LPN group and 92% and 89% for the LCA group.[33]

### Hilar tumors

Small renal masses in close proximity to the renal hilum pose a challenge to surgical management using nephron-sparing approaches. Hruby and co-workers retrospectively compared patients with hilar tumors who had undergone LCA and LPN. Laparoscopic partial nephrectomy was performed using a transperitoneal or retroperitoneal approach in twelve patients, and laparoscopic cryoablation was completed on eleven patients. Patients were followed at regular intervals with a contrast enhanced CT scans. Immediate outcome variables including operative time, estimated blood loss, hospital stay, and post-operative analgesic requirement were compared in the two groups. Laparoscopic cryoablation was associated with a faster operative time, reduction in blood loss, shorter hospital stay and decreased amount of narcotic pain medication compared with LPN. In addition, no intra-operative or postoperative complications occurred in the LCA cohort compared to nine complications in the LPN group, which included four urinary fistulas. Although LCA is difficult to perform in patients with hilar lesions, it is a reasonable option for tumors in this location and is associated with better immediate surgical outcomes and fewer complications compared with LPN.[34]

## FOLLOW-UP IMAGING AND BIOPSY

Both CT and MRI are commonly used for tumor surveillance after cryoablation. Immediately after ablation, the lesion appears larger than the original tumor, which occurs because the ice ball created with cryoablation extends well beyond the visible margins of the targeted tumor. At follow-up examination, ablated renal tumors are seen as focal masses without contrast enhancement that frequently decrease in size. However, in the short-term, there may be an apparent tumor size increase due to perinephric stranding and fibrosis at the margin of the ablation zone limiting the ability to precisely interpret the ablation site boundaries. Clinical follow-up for patients after cryoablation was reported in a recent multi-institutional study recommending postoperative imaging using either renal protocol CT or MRI at one, three or six, and 12 months after treatment. New enhancement or enlargement of the ablation zone is suspicious and should be considered for biopsy.[30]

Gill and colleagues reported a positive biopsy for RCC in two patients undergoing a six-month post-ablation evaluation. Both patients had no evidence of enhancement or enlargement of the tumor bed on MRI. The remaining 37 patients biopsied at six months demonstrated fibrosis, necrosis and irreversible cell death with no evidence of RCC.[22] Biopsy results of post ablation tumor sites at six months demonstrated favorable results in 192 consecutive renal lesions treated with LCA. Follow-up imaging with CT scan was performed in 72% of the patients at the time of biopsy. Radiographic success defined as no central or nodular enhancement on contrast CT occurred in 90% of cases. Six positive biopsies were found all consistent with some form of radiographic enhancement. All 60 patients without evidence of post-ablation enhancement had negative biopsies.[35]

## NOVEL LAPAROSCOPIC AND MINIMALLY INVASIVE TECHNIQUES

In recent years, two unique methods of access to perform renal cryoablation have been reported: Single Port Access Renal Cryoablation (SPARC) and Natural Orifice Transluminal Endoscopic Surgery (NOTES). Single Port Access Renal Cryoablation uses a novel multichannel single port to introduce laparoscopic instruments through. The port is placed at the umbilicus in transperitoneal approaches, and at the tip of the 12th rib for a retroperitoneal approach. Goel and co-workers reported results in six patients undergoing SPARC (two transperitoneal and four retroperitoneal) for renal masses with a mean size of 2.6cm. All procedures were performed successfully without laparoscopic or open conversion and there were no intra-operative complications. Estimated blood loss was 86mL and one patient required transfusion post-operatively.[36] Difficulties with this approach include a limited range of motion with surgical instruments and frequent instrument fencing. New second generation flexible instruments with articulating capability have improved the range of motion and the ability to maneuver multiple instruments through the single port. Five patients underwent single port laparoscopic retroperitoneal surgery (SPLRS) with cryoablation and the results were retrospectively compared to a matched cohort of patients who underwent LCA via a retroperitoneal approach. Mean tumor size, OR time, and EBL were 2.34cm, 174min, and 75mL respectively. Comparison with LCA demonstrated no statistical difference in these parameters. However, patients receiving SPLRS cryoablation had statistically significantly lower visual analog (VAS) pain scores measured at hospital discharge compared to the LCA cohort.[37]

NOTES is being investigated as a way to further reduce patient morbidity by improving post-operative pain and reducing post-operative scar formation. The natural orifice approach consists of a flexible endoscope to enter the peritoneal cavity. A double channel endoscope is used to provide entry points for instruments. Experience with transgastric and transvaginal NOTES cryoablation in a porcine model resulted in successful completion of the procedure without the need for laparoscopic port placement. Access to kidney was obtained and cryoablation was performed on the anterior aspect of the upper pole in each porcine kidney. Operative time was faster for the transvaginal compared to the transgastric approach (74min vs. 91min). There were injuries to the kidney or adjacent organs.[38]

NOTES and SPARC are the next phase in the evolution of minimally invasive surgery and will be used with current cryoablative technology and nephron sparing surgery techniques for the treatment of small renal masses.

## FUTURE TRENDS

Renal cryoablation continues to be modified and refinement of current techniques and instruments are anticipated. Further, as long-term data accumulates cryoablation will be established as an attractive option for the treatment of small renal masses leading to an increase in the number of patients treated with this modality. Technologic advances in cryoprobes, more effective computer models to estimate tumor volume, and better models to predict tissue response to cryoablation are being researched. New probes are being developed to monitor voltage differentials across cell membranes, as cancerous tissue conducts voltages differently than normal tissue due to alterations of cell membranes.[39] These probes may provide information at surgical margins that helps distinguish normal and cancerous tissue. The addition of advanced computer modeling for estimation of tumor volume with 3-D bubble packing algorithms may lead to a new understanding of surgical margins.[40,41] Improvement in intra-operative imaging such as electrical impedance tomography (EIT) provides visualization of the ice-front formed by the cryoprobe; this may allow for a more precise cryoablation with better control of surgical margins.[42–44]

## ALTERNATIVE ABLATIVE THERAPIES

### Radiofrequency ablation

Radiofrequency ablation occurs by the transfer of high-frequency electrical current into target tissue culminating in thermal energy. Temperatures in excess of 60^C^ cause tissue destruction through coagulative necrosis, fibrosis, and thermally induced vascular thrombosis.[45] The conductive heat spreads to adjacent tissue leading to tissue ablation.[46] Similar to cryoablation, the ideal ablation zone for renal tumors treated with RFA is 1 cm beyond the tumor margin based on pre-operative CT or MRI. RFA and ablation zone diameter can be monitored using either impedance or temperature-based generators.[47] Impedance systems indicate tumor ablation when tissue adjacent to the probe(s) demonstrates infinite impedance (ohms). This implies complete dessication and charring suggesting that electric current is unable to pass through tissue. Levels ≥ 200 Ω are recommended (Radiotherapeutics, Boston Scientific, Natick, Massachusetts, USA) for proper tumor ablation. When tissue reaches this level of impedance, the ablation zone diameter stabilizes. However, when charred tissue becomes adherent to the ablation probes an artificial increase in impedance occurs, which limits the ablation zone. Changes in probe design have led to the concept of “wet RFA”, which consists of cool saline irrigation that eliminates the charred tissue from the probe and allows for more efficient heat transfer.[45]

In addition, RFA can be monitored with temperature-based systems, which functions by allowing probes to heat to a specified pre-set temperature for a predetermined length of time. One issue to consider when using this monitoring system is that there can be a discrepancy between the probe and tissue temperatures, which may affect the degree of cellular destruction necessary for proper tumor ablation.[45] Similar to cryotherapy, radiofrequency ablation may also be performed with an open, laparoscopic or percutaneous approach. Tumor characteristics and patient preference dictate the final method of treatment. Posterior tumors are usually managed percutaneously and anterior tumors are treated laparoscopically. For laparoscopic RFA, intraoperative ultrasound is used to identify the tumor location, however with a percutaneous approach a pre-operative CT is used for tumor localization. Unlike cryoablation, the status of the ablation process during RFA cannot be monitored in real time with any imaging modality. Therefore, the CT or MRI obtained pre-operatively is used to determine factors such as generator temperature, duration of ablation, and number of ablation cycles. Percutaneous procedures can be accomplished under conscious sedation instead of general anesthesia, which improves the minimally invasive nature of the procedure and allows for the surgery to be performed on an outpatient basis.

### Outcomes with radiofrequency ablation

In a multi-institutional study, an initial ablation rate of 97% was observed after percutaneous RFA. Overall one and three year recurrence-free survival was 97% and 92% respectively.[48] At this time, there are no studies in the literature with long-term oncologic follow-up after percutaneous RFA. Levinson and co-workers presented long-term follow-up on thirty-one high-risk surgical patients who underwent percutaneous RFA with a mean lesion size was 2.0 cm (1.0-4.0 cm). After a mean follow-up of 61.6 months, the recurrence-free, cancer-specific, and overall survival rates were 90.3%, 100%, and 71% respectively. There was one failure, which was successfully re-ablated with RFA. Three patients recurred at 7, 13, and 31 months and were treated with repeat RFA, PCA, an laparoscopic radical nephrectomy respectively.[49] Although laparoscopic RFA is well described, there are no studies with long-term follow-up. Park and colleagues reported data on 94 renal tumors (39 performed laparoscopically), with a mean tumor size of 2.4 cm. At a mean follow-up of 25 months, the cancer-specific and overall survival rates were 98.5% and 92.3% respectively.[50]

### High-intensity focused ultrasound

High-intensity focused ultrasound (HIFU) is an extracorporeal technique that treats renal tumors with a focused ultrasound wave that passes through the body to its target at a selected depth and is converted to heat energy. Piezoelectric generators are used to control ablation volume by adjusting the power, duration, and location of the ultrasound waves. Renal tumors are targeted with ultrasound prior to ablation, however real time monitoring of the progression of the ablation cannot be done.[51] Common side effects include treatment site discomfort, skin burns, and fever. At this time there is limited data available for HIFU and renal tumors. Hacker et al reported on 19 patients with renal tumors who underwent nephrectomy. Prior to surgery patients were treated with HIFU lesions on normal parenchyma to the tumor bearing kidney. On final pathology, fifteen tumors had evidence of variable sign of tissue ablation. In addition, there was no correlation with the energy level and the lesion size never reached the targeted volume.[52] Illing and coworkers reported an early study on eight patients treated with HIFU. Four patients had radiologic confirmation of tumor ablation, however only 1 out of 4 patients undergoing histological evaluation, had evidence of thermal damage and this area was smaller than originally targeted.[53]

Certain technical disadvantages of HIFU include lack or real time monitoring of tissue ablation, poor acoustical interphases between the transducer and the abdominal wall, and issues with patient/kidney movement secondary to respiration.[51] Laparoscopic HIFU was introduced to help combat problems with movement and interphases. Klingler and colleagues reported findings on ten patients who underwent laparoscopic HIFU for renal masses. Two patients with 9 cm tumors underwent HIFU on an external area of the tumor to prove feasibility of technique and then immediately underwent radical nephrectomy. In these patients, both marker lesions demonstrated homogeneous thermal damage. Eight patients with a mean tumor size of 2.2cm underwent ablation with HIFU for curative intent. The ablation included a 2-3mm margin of normal parenchyma. Seven patients proceeded to laparoscopic partial nephrectomy to evaluate the extent and efficacy of ablation. Four of these tumors showed complete ablation of the entire tumor. Two tumors had a 1-3mm rim of viable tissue and one tumor had a central area of viable tissue present, which corresponded to approximately 20% of the tumor volume. One patient did not undergo partial nephrectomy after ablation. Core biopsies were taken immediately following treatment showing severe thermal damage. Follow-up CT scans at 3 and 6 months have shown shrinkage of the lesion and no enhancement.[54]

## CONCLUSIONS

The treatment of small renal masses has transitioned from radical nephrectomy to nephron sparing surgery. Technology advanced the management of small renal masses further with the use of laparoscopic and robotic partial nephrectomy. Currently, laparoscopic cryoablation and percutaneous cryoablation represent the most recent addition in the continuous evolution of minimally invasive surgery techniques.

Renal cryoablation is emerging as the preferred ablative procedure for patients with small renal masses ≤ 4cm. Indications for the procedure, in addition to size, are patients who are at high surgical risk due to prior abdominal surgery or significant existing co morbidities and patients who reject extirpative surgery. It is also a good option for patients with an anatomic or functional solitary kidney or multiple renal tumors. Intermediate to long-term outcomes from the procedure suggest that cryoablation is a safe alternative to laparoscopic partial nephrectomy providing short-term patient benefits such as a shorter hospital coarse, reduced post-operative pain, and a decreased rate of complications. Urologists must be familiar with renal cryoablation to adequately treat and council patients presenting with clinical T1a renal tumors.
